# Modeling neural contrast sensitivity functions in human visual cortex

**DOI:** 10.1162/imag_a_00469

**Published:** 2025-02-18

**Authors:** Carlien Roelofzen, Marcus Daghlian, Jelle A. van Dijk, Maartje C. de Jong, Serge O. Dumoulin

**Affiliations:** Spinoza Centre for Neuroimaging, Amsterdam, Netherlands; Computational Cognitive Neuroscience and Neuroimaging, Netherlands Institute for Neuroscience, Royal Netherlands Academy of Arts and Sciences, Amsterdam, Netherlands; Experimental and Applied Psychology, Vrije Universiteit Amsterdam, Amsterdam, Netherlands; Laboratory for Experimental Ophthalmology, University Medical Center Groningen, Groningen, Netherlands; Experimental and Applied Psychology, University of Amsterdam, Amsterdam, Netherlands; Experimental Psychology, Helmholtz Institute, Utrecht University, Utrecht, Netherlands

**Keywords:** contrast sensitivity function, contrast response function, spatial frequency, visual cortex, fMRI

## Abstract

The contrast sensitivity function (CSF) characterizes visual function, and iswidely used in research on visual perception and ophthalmological disorders. TheCSF describes the lowest contrast level that participants can perceive as afunction of spatial frequency. Here, we present a new method to estimate theneural equivalent of the CSF that describes how a population of neurons respondsto contrast as a function of spatial frequency. Using functional magneticresonance imaging (fMRI) at 7 Tesla, we measured neural responses whileparticipants viewed gratings that varied systematically in contrast and spatialfrequency. We modeled the neural CSF (nCSF) using an asymmetric parabolicfunction, and we modeled the transition from no response to full response usinga contrast response function (CRF). We estimated the nCSF parameters for everycortical location by minimizing the residual variance between the modelpredictions and the fMRI data. We validated the method using simulations andparameter recovery. We show that our nCSF model explains a significant amount ofthe variance in the fMRI time series. Moreover, the properties of the nCSF varyaccording to known systematic differences across the visual cortex.Specifically, the peak spatial frequency that a cortical location responds todecreases with eccentricity and across the visual hierarchy. This new methodwill provide valuable insights into the properties of the visual cortex and howthey are altered in both healthy and clinical conditions.

## 1 Introduction

The detection of contrast and spatial frequency are fundamental aspects of vision.These features are important for object recognition and essential in everyday life.Where contrast is important in distinguishing an object from its background, spatialfrequency reflects object size (Chung& Legge, 2016). The ability to detect a certain contrast is givenby contrast sensitivity (100/contrast threshold) and depends on spatial frequency(Campbell & Robson,1968;Lesmes et al.,2010;Sowden et al.,2002). The relation between contrast sensitivity and spatial frequency isdescribed by the contrast sensitivity function (CSF). Behaviorally, the CSF definesthe lowest contrast one can perceive as a function of spatial frequency (Campbell & Robson, 1968),thereby defining the threshold between the visible and invisible (Pelli & Bex, 2013).

The CSF is used to assess visual function. For example, the CSF is affected in manyophthalmological conditions, including amblyopia (Howell et al., 1983;Koskela & Hyvarinen, 1986;Sjöstrand, 1981;Wang et al., 2017), maculardegeneration (Kleiner et al.,1988), optic neuritis (Zimmern et al., 1979), glaucoma (Ichhpujani et al., 2020), retinitis pigmentosa (Hyvärinen, 1983), cataract(Vasavada et al., 2014), andcorneal edema (Hess & Garner,1977). Additionally, the CSF can be altered in neurological conditionssuch as multiple sclerosis (Regan etal., 1981), cerebral lesions (Milling et al., 2014), Parkinson’s disease (Ridder et al., 2017), andschizophrenia (Cimmer et al.,2006).

In general, the CSF correlates with visual acuity (Hou et al., 2010;Stalin & Dalton, 2020). However, the CSF measuresthe detection of a stimulus using a wide range of contrasts and spatial frequencies,and has therefore been proposed as a more suitable tool to assess visual performancecompared to standard visual acuity tests (Huang et al., 2007). For example, the CSF measures visualdeficits missed by standard visual acuity tests. Thus, the CSF provides deeperinsights regarding functional vision and improves detection of visual pathology(Huang et al., 2007;Lesmes et al., 2010).

The CSF characterizes visual perception and is altered by changes in the eye as wellas changes in neural processing. In healthy participants, the CSF can be altered bycognitive manipulations such as attention, for example, contrast sensitivity isincreased for attended stimuli and decreased for unattended stimuli (Pestilli et al., 2007).Furthermore, the CSF changes across development, during infancy as well as latechildhood (Dekker et al., 2020).In addition, several visual disorders are at least, in part, caused by neuraldeficits, for example amblyopia (Barrettet al., 2004) and glaucoma (Murphy et al., 2016). This highlights the relevance of studying neuralprocesses underlying the CSF, in addition to the ocular components. Thus, a neuralmeasure of the CSF would expand our understanding of how cortical processing isaltered with cognition and different visual disorders.

Here, we translate the CSF from psychophysics, by introducing a new method thatestimates the neural CSF (nCSF) in the human visual cortex using fMRI. The nCSFconcept is derived from ophthalmology where it refers to the ability of the retinatogether with the brain to resolve an image, that is, not affected by eye-optics(Campbell & Gubisch,1966;Le Grand, 1935;Michael et al., 2011). ThenCSF approach builds on previous studies showing that individual neurons in thevisual cortex are sensitive to contrast and spatial frequency (Albrecht & Hamilton, 1982;Levitt et al., 1994;Sclar et al., 1990). Thissensitivity is also present at the neural population level with fMRI studies showingsystematic changes in contrast (Boyntonet al., 1999;Marquardt etal., 2018), spatial frequency (Aghajari et al., 2020;Broderick et al., 2022;Henriksson et al., 2008;Singh et al., 2000;Sirovich & Uglesich, 2004), and their combination(Goulet & Farivar,2024). Here, we combine population measures of the CSF (akin to thepopulation receptive field (pRF) method;Dumoulin & Wandell, 2008) and we model thetransition from no response to full response, by varying the slope of the contrastresponse function (CRF,Boynton et al.,1999).

We show that our nCSF model effectively captures the variance in the fMRI timeseries. Moreover, the properties of the nCSF vary systematically with eccentricity,polar angle and across the visual hierarchy. Overall, we describe a quantitativevalidated framework to model nCSF properties.

## 2 Methods

### 2.1 Participants

We present data from five participants (two males, age range 26-45 years). Allparticipants had normal or corrected-to-normal visual acuity, as confirmed bytesting visual acuity using a tumbling E eye chart. The participants gavewritten informed consent prior to the start of the experiment. The study wasapproved by the Ethical Committee of Vrije Universiteit Amsterdam in accordancewith the World Medical Association’s Declaration of Helsinki.

### 2.2 Stimulus presentation

The stimuli were generated using PsychToolbox (Brainard, 1997;Pelli, 1997) in MATLAB (version R2018b, Mathworks).Participants viewed the stimuli on a gamma corrected 32-inch BOLD screen display(Cambridge Research, 1920 x 1080 pixels), through an angled mirror attached tothe head coil (distance 220 cm). 10-bit mode was enabled on the BOLD screendisplay to facilitate presentation of stimuli with contrasts below 0.78%Michelson contrast. Participants were instructed to fixate on a dot in thecenter of the screen, and to press a button when the dot changed color.

**Fig. 1.**
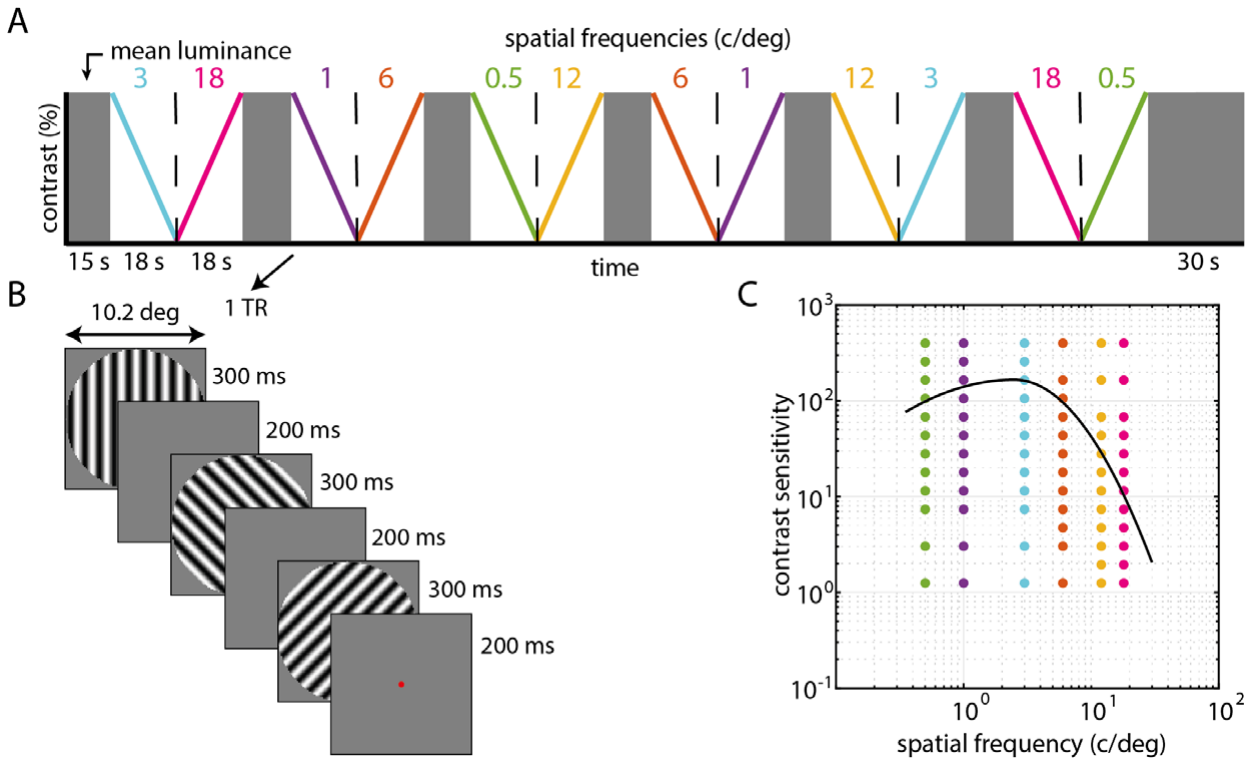
Schematic representation of the stimulus design. (A) We presentedfull-field static sinewave gratings with six different spatialfrequencies (0.5, 1, 3, 6, 12, 18 c/deg). Gratings with the same spatialfrequency are presented in both descending contrasts and ascendingcontrasts in a fixed semi-random order. (B) During every MRI volumeacquisition (1.5 s), three gratings with the same contrast but adifferent orientation were shown. Each grating presentation lasted for300 ms followed by mean luminance lasting 200 ms. The participantsfixated the red dot. (C) A typical CSF curve with dots showing thestimulus sampling grid across spatial frequency and contrast sensitivity(100/contrast).

The nCSF stimulus consisted of different static sinewave gratings with a circularaperture of 10.2 degree diameter of visual angle and raised cosine edge,presented on a mean luminance background. The gratings varied systematically incontrast and spatial frequency; seeFigure 1. Each stimulus block lasted 18 s and consisted of gratingswith the same spatial frequency but systematically changing (either decreasingor increasing) in contrast (Fig.1A). Three different gratings were shown every 1.5 s with the samecontrast but a different orientation and phase (Fig. 1B), to ensure that the stimulus wasrefreshed on the retina. Twelve different contrasts were used in each stimulusblock, ranging from 0.25–80% Michelson contrast. An increasing anddecreasing contrast block was included for each spatial frequency (0.5, 1, 3, 6,12, 18 c/deg), and the order in which these blocks were presented was fixed; seeFigure 1A. We includedstimulus blocks with both increasing and decreasing levels of contrast to avoiddirectional effects introduced by the HRF (similar as in pRF designs), and toavoid the threshold being dependent on whether the contrast change is increasingor decreasing.

Before every two stimulus blocks there was a mean luminance block lasting for 15s, and there was an extra 15 s mean luminance block in the end. The orientationof the gratings was randomized, and two subsequent gratings had at least a45-degree difference in orientation. Each grating presentation lasted for 300 msand was followed by a 200 ms presentation of mean luminance (seeFig. 1B). The total stimulusprotocol lasted 321 s per run. The contrast range presented was dependent on thespatial frequency shown in the stimulus block, ensuring an optimal sampling ofthe CSF (seeFig. 1C, andTableS1).

Retinotopic (pRF) data were collected as part of previous experiments; thestimuli consisted of a drifting bar, moving in eight directions. For four of theparticipants, the texture inside the bar comprised natural images. For the fifthparticipant, the bar contained flashing checkerboards. Stimuli were presentedusing the same screen (without the 10-bit mode) and software as in the nCSFexperiment.

### 2.3 Data acquisition

Anatomical and functional magnetic resonance imaging (fMRI) data were acquired ona 7 Tesla Philips Achieva scanner (Philips, Best, Netherlands) with an 8-channelMultiX head coil. Anatomical images were collected using a 3D MP2RAGE sequence(TR = 6.2 ms, TE = 3.0 ms, flip angle = 5.0 degrees, FOV= 220 x 220 x 164 mm, voxel size = 0.7 mm isotropic) (Marques et al., 2010).

fMRI data were acquired using a T2*-weighted 2D echo-planar imaging (EPI)sequence (TR = 1.5 s, TE = 22.5 ms, flip angle = 65degrees, FOV = 216 x 216 x 97 mm, 57 slices, voxel size = 1.7 mmisotropic) oriented across the visual cortex. For each participant, 10–12functional scans were acquired, with an approximate duration of 5 min perfunctional run. Each functional run was followed by a TOPUP scan in order tocorrect for local image distortions. Additionally, pRF data were collected forone participant (8 functional runs) with the same sequence for visual fieldmapping (seeSection 2.6). Forthe remaining four participants, pRF mapping data were already available.

### 2.4 Data pre-processing

For the anatomical data, the MP2RAGE sequence was used to obtain a T1-weightedanatomical image, resulting in a set of different gradient echo images, that is,the first inversion (T1-weighted image) and a second inversion (proton densityscan). The first and second inversion were combined to generate a singleanatomical image, corrected for proton density. The anatomical image wasskull-stripped using the*3dSkullstrip*function in AFNI (Cox, 1996). Segmentation ofgray and white matter was performed using CBS Tools in MIPAV (Bazin et al., 2007). Thecortical surfaces for each participant were reconstructed using the FreeSurfer7.2*recon-all*function (Dale, 1999). To improve the FreeSurferreconstructions particularly around the sinus, segmentations from CBS Tools wereadded to Freesurfer’s*“brainmask.mgz”*file, and*recon-all*was run again.

For the functional data, all functional images were pre-processed using AFNI. Thewarp field of all functional images was estimated based on the EPI and TOPUP ofeach run to correct for susceptibility distortions. The motion parameters werecalculated to apply motion correction to all functional images. Then, all warpedand motion corrected volumes were combined to generate an average EPI image.This EPI image was registered to the anatomical image. The volume of the meanEPI image was masked using the AFNI function*3dAutomask*toreduce volume size, and was zero-padded using*3dZeroPad*. Thecenter of mass of the anatomical volume was aligned to the mean EPI image using*@Align_centers*to improve co-registration. Then, the meanEPI image was manually shifted and rotated using the*Nudgedataset*AFNI plugin, after which the automated registrationfunction*3drotate*optimized the co-registration using affinetransformation. The co-registration was then applied to all EPI images of allindividual functional runs using the function*3dNwarpApply*withnearest-neighbor interpolation. The co-registered functional volumes were thenprojected to the participant’s cortical surface using the*mri_vol2surf*function in FreeSurfer (Dale, 1999). Veins wereidentified by displaying the mean EPI signal on the cortical surface, andmasking those vertices with relatively low signal intensity. The BOLD timeseries were then detrended by demeaning, applying the discrete cosine transform(DCT), and removing the first three DCT coefficients to eliminate low-frequencytrends. The detrended data were further processed by averaging over functionalruns and converting to BOLD percent signal change.

### 2.5 Model-based analysis

**Fig. 2.**
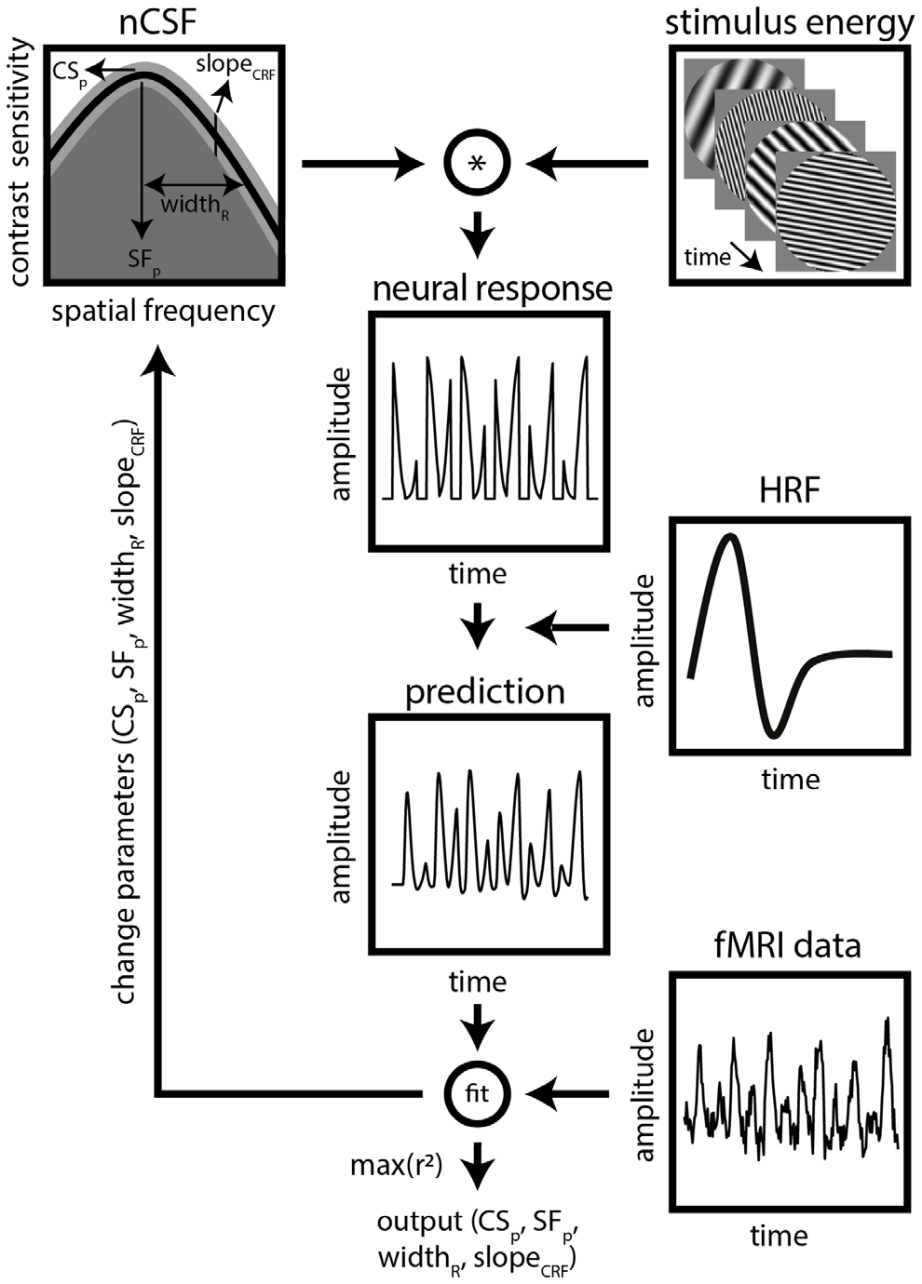
Schematic overview of the nCSF model-based analysis. The nCSF modelcombines the CSF (Chung& Legge, 2016) with the CRF (Boynton et al., 1999).We predict the fMRI response by a multiplication of the nCSF model withthe stimulus sequence, and convolve this model time course with the HRF.We vary the slope of the CRF (*slope_CRF_*) andthe CSF parameters (*CS_p_*,*SF_p_*, and*width_R_*). The optimal model parametersare estimated by maximizing the variance explained(*r^2^*) between the predicted andmeasured fMRI time series, similar to the pRF method (Dumoulin & Wandell,2008).

The nCSF model combines the contrast sensitivity function (CSF) with the contrastresponse function (CRF) and aims to fit the CSF in each cortical location(voxel), thereby obtaining the nCSF (Fig. 2). The CSF is described byChung and Legge (2016)and is characterized as anasymmetric parabolic function with the following equation:

$$f\left(SF\right)=\{\begin{array}{c} CS_{p}-{\left(SF-SF_{p}\right)}^{2}\times {\left(width_{L}\right)}^{2}ifSF<SF_{p},\\ CS_{p}-{\left(SF-SF_{p}\right)}^{2}\times {\left(width_{R}\right)}^{2}ifSF\geq SF_{p} \end{array}$$ (Equation 1)

where*f*(*SF*) is the contrast sensitivity atspatial frequency (*SF*),*CS_p_*is thepeak contrast sensitivity,*SF_p_*is the spatialfrequency at which*CS_p_*occurs (peak spatialfrequency), and*width_L_*and*width_R_*are the curvatures of the left and rightbranches of the asymmetric parabolic function, respectively (Chung & Legge, 2016).Note, following the notation inChung and Legge (2016), “*width”*parameters refer to the “fall off rate”, hence a CSF with a high*width_r_*actually has a narrower shape (thereis a faster drop in sensitivity to higher spatial frequencies). The*width_L_*is set at 0.68 (Chung & Legge, 2016),and*width_R_*is varied as a model parameter, since weexpect the most variance on the right branch at higher spatial frequencies(*SF*>*SF_p_*). We reportthe normalized area under the log contrast sensitivity function(*AUC*, %) as a summary metric of the full CSF for spatialfrequencies between 0.5–18 c/deg (stimulus space).*AUC*is calculated by approximating the integral of the logarithmic CSF, andnormalizing it with reference to the area under a “standard”logarithmic CSF calculated from healthy controls (from Chung & Legge,2016:*CS_p_*= 166,*SF_p_*= 2.5 (c/deg),*width_L_*= 0.68,*width_R_*= 1.28, shown inFig. 1C).

The parameters*SF_p_*and*CS_p_*are transformed to log_10_spacebefore fitting the CSF (Equation 1,Chung & Legge, 2016). Here, we used an adapted version ofEquation 1whereall parameters are defined in linear space before fitting the CSF:

$$f\left(SF\right)=\{\begin{array}{c} 10^{log_{10}\left(CS_{p}\right)-(log_{10}\left(SF\right)-\left(log_{10}\left(SF_{p}\right)\right)\times {\left(width_{L}\right)}^{2}}ifSF<SF_{p},\\ 10^{log_{10}\left(CS_{p}\right)-(log_{10}\left(SF\right)-\left(log_{10}\left(SF_{p}\right)\right)\times {\left(width_{R}\right)}^{2}}ifSF\geq SF_{p} \end{array}$$ (Equation 2)

For transforming the binary response of the CSF to a gradual response, we addedthe CRF to the nCSF model (Fig.2). We fitted CRFs using the following equation (modified fromBoynton et al. (1999)):

$$R\left(C\right)=a\frac{C^{q}}{C^{q}+Q^{q}}$$ (Equation 3)

where*R*is the fMRI response and*C*is theamount of RMS-contrast. The variables for*Q*and*q*define the shape of the CRF.*Q*represents the contrast where the fMRI response is at 50%, and was depended onthe CSF. We fitted the variable*q*or*slope_CRF_*.

The nCSF parameters were estimated from the fMRI data using a model-based fittingapproach (Fig. 2), similar tothe population receptive field (pRF) method (Dumoulin & Wandell, 2008). First, the fMRIblood oxygen level dependent (BOLD) response is predicted through multiplyingthe nCSF model with the stimulus sequence and convolving the time course withthe hemodynamic response function (HRF). Here, the HRF was modeled using the twogamma basis functions (Glover,1999). The coefficients corresponding to the canonical HRF, itsderivative, and dispersion were fixed at values of 1, 1, and 0, respectively(Pedregosa et al.,2015). The same HRF was used in fitting both the nCSF and pRF model. Wevaried the model parameters (*CS_p_, SF_p_,width_R_*and*slope_CRF_*) ina coarse-to-fine manner. All model parameters were varied until the residualsum-of-squares (*RSS*) is minimized between the predicted andmeasured fMRI data, thereby maximizing the variance explained(*r^2^*) of the model. The nCSF model was fittedto all cortical locations (voxels) independently. We selected cortical locationswith*r^2^*> 30% in both pRF and nCSF model fitsfor further analyses.

### 2.6 Region of interest definition

For the region of interest (ROI) definition, pRF mapping was used (Dumoulin & Wandell,2008). These data were already available for four participants, andwe collected pRF data for one participant (seeSection 2.3). We included visual field maps V1,V2, V3, hV4 and visual field map clusters TO, LO, and V3AB as ROIs (Wandell et al., 2007).

### 2.7 Model validation

We validated the method using simulations (Dumoulin & Wandell, 2008;Lerma-Usabiaga et al., 2020),where the ground truth is known, and estimated the accuracy through modelparameter recovery. To validate the nCSF method, we simulated two nCSF modelswith different CSF parameters (in green:*SF_p_*= 1 c/deg,*CS_p_*= 150,*width_R_*= 1.3, in red:*SF_p_*= 2 c/deg,*CS_p_*= 100,*width_R_*= 1). For each simulated dataset,the predicted response was calculated using the stimulus sequence (as describedinSection 2.2) andcombination of model parameters, convolved with the standard HRF. We normalizedthese synthetic voxel time series and added three different noise levels. Noisewas generated by sampling from a normal distribution (*M*= 0,*SD*= 1) multiplied by a scaling factor 0.4,0.7, or 1.1. For each noise level and combination of model parameters, 100 timeseries were generated. Next, we fitted the nCSF model using the same HRF usedfor the main data analysis (seeSection 2.5). The model parameters were extracted from the simulateddatasets, and the variance explained for each of the three noise levels wascomputed, resulting in the low, medium, and high variance explained categories.The two different nCSF curves for the three variance explained categories areshown inFigure 3.

**Fig. 3.**
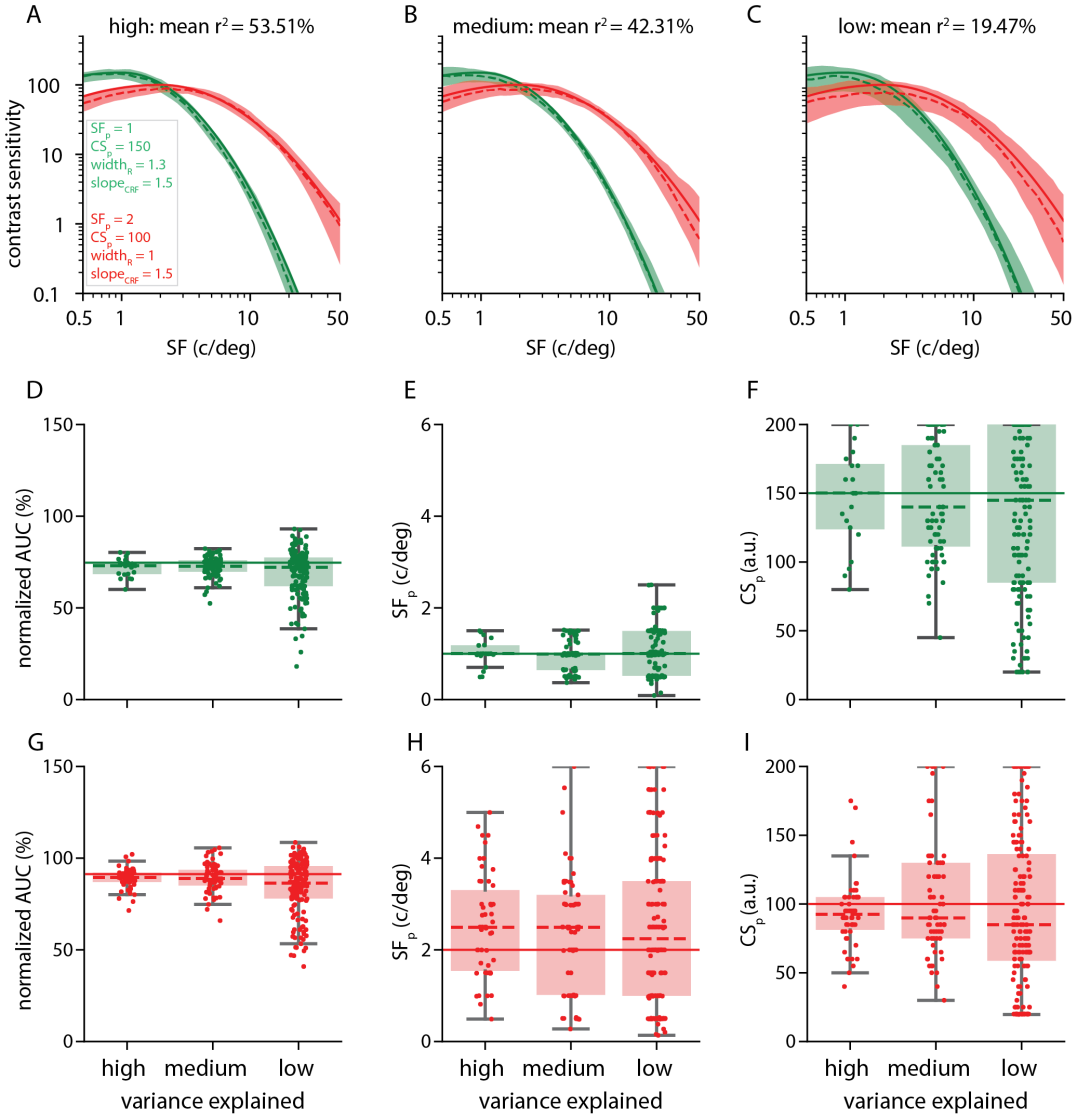
nCSF model validation. We tested different combinations of nCSF modelparameters, resulting in different nCSF curves (panels A-C). For eachcombination of parameters, the results are shown for synthetic data (100permutations) and three variance explained categories (high:*r^2^*> 50%; medium: 30%<*r^2^*< 50%; and low: 10%<*r^2^*< 30%, left to right,respectively). The solid lines represent the nCSF curves based on thechosen parameters, the dashed lines represent the median nCSF curves,and the shaded areas represent the 25th and 75th percentile. Panels(D-I) Show how well the parameters are recaptured: solid lines indicatethe true nCSF model parameters, whereas the dashed lines anddistributions represent the median values of 100 permutations. For eachmodel parameter the effect is shown for the high, medium, and lowvariance explained category (left to right, respectively). (D) and (G)Normalized*AUC*(%, output variable). (E) and (H)*SF_p_*(c/deg). (F) and (I)*CS_p_*(a.u.). Overall, the nCSF fitrecovers the parameters, but the variability increases with increasingnoise (lower variance explained). Some parameters, in particularnormalized*AUC*(%, output variable) and*SF_p_*(c/deg)*,*aremore stable than others (in particular*CS_p_*).

Additionally, we simulated the effect of the HRF on the model parameters (see HRFSimulation section inSupplementary Materials). Furthermore, wesimulated how*slope_CRF_*varies with noise, and theeffect of slowing speed at which the stimulus changes contrast (see CRFSimulation section inSupplementary Materials).

## 3 Results

### 3.1 Validation of the nCSF model-based analysis

Validation is essential for method development (Aqil et al., 2021;Dumoulin et al., 2003;Dumoulin & Wandell, 2008). Moderndata-analyses techniques are complex and it is impossible to check the softwareby eye: ground-truth datasets are needed (Lerma-Usabiaga et al., 2020). Here, we validated thenCSF model using simulations.

We created two “ground-truth” nCSF models, with differentparameters (red and green inFig.3), added noise, and refitted the resulting time series to determinehow well we can recapture the model parameters (seeSection 2). We splitted the resulting fits intothree variance explained levels: high (*r^2^*>50%), medium (30% <*r^2^*< 50%), and low(10% <*r^2^*< 30%). Across all varianceexplained levels (seeFig.3A-C), the median estimated nCSF curves (dashed lines in red andgreen) closely match the true curve (solid lines in red and green). However, thespread of the estimated nCSF curves increased with lower levels of varianceexplained (compare shaded regions inFig. 3A-C). The median values of the recovered parameters closelymatch the ground-truth values (compare the dotted with solid horizontal lines inFig. 3D-I). Again, thespread of the recovered parameters increases from high to low variance explainedlevels (seeFig. 3D-I).

### 3.2 nCSF model captures cortical responses across the visual hierarchy

**Fig. 4.**
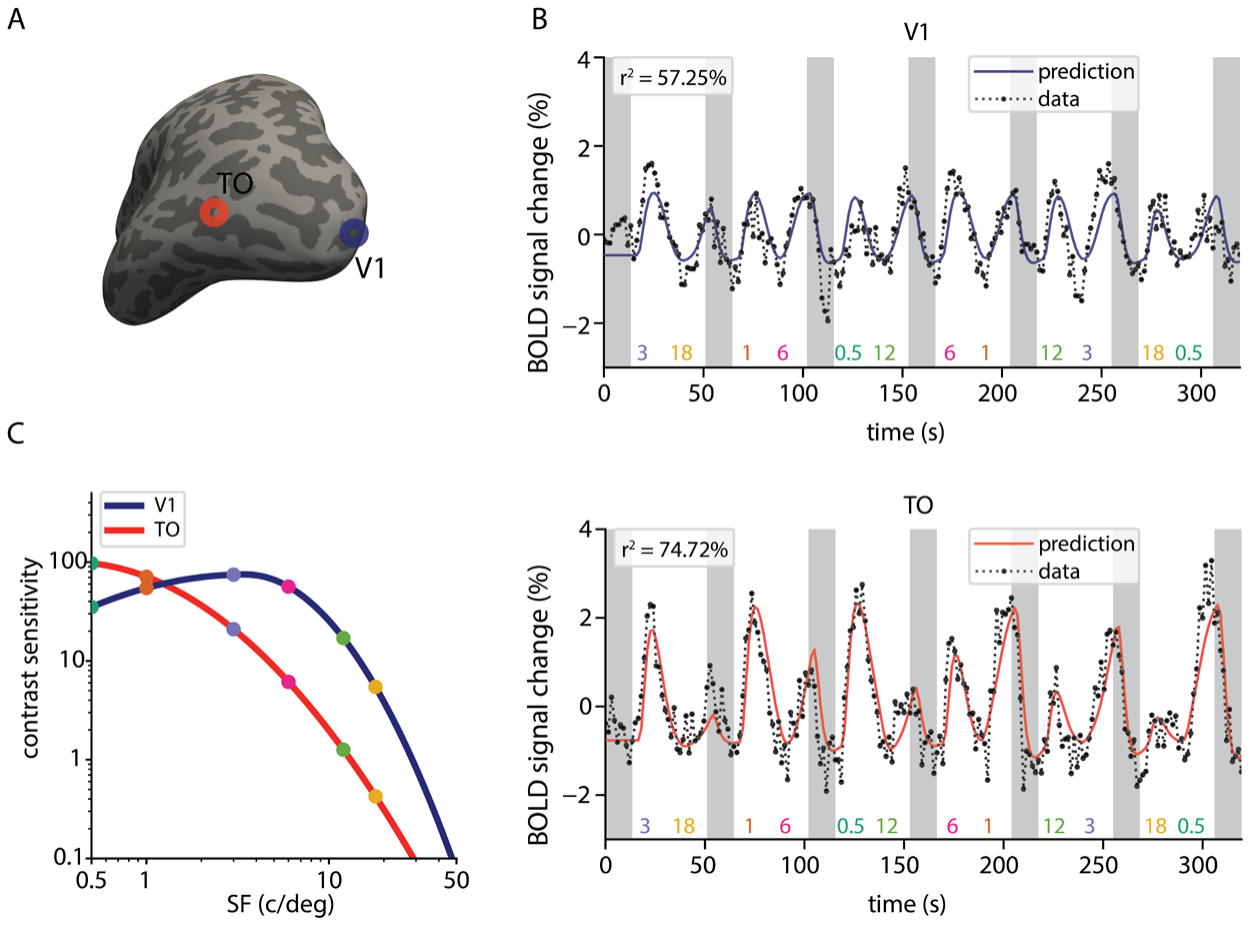
Example fMRI time series and nCSF model fits. (A) Inflated corticalsurface (left hemisphere) showing the location of the example corticallocations in V1 (blue circle) and TO (red circle). (B) Predicted (solidline) and actual (dotted line) fMRI time series of a vertex in V1 (toprow) and TO (bottom row). (C) nCSF curves for example cortical locationsin V1 (blue) and TO (red). Each dot represents one of the spatialfrequencies used in the stimulus sequence.

We fitted the nCSF model to the fMRI time series across the cortex, demonstratingits ability to capture a diverse range of responses.Figure 4presents example fits from individualvertices in the V1 and TO regions (Fig. 4A), highlighting contrasting parameter values. The varianceexplained by the model for these locations was 74.72% for TO and 57.25% for V1,indicating that the nCSF model effectively captures the variance in the fMRItime series across both regions. Furthermore, the predicted fMRI time seriesclosely align with the recorded data, as illustrated inFigure 4B. Notably, the nCSF curve in V1exhibits a higher*SF_p_*compared to the corticallocation in TO (Fig. 4C).

### 3.3 nCSF properties vary across the cortex

Here, we report the parameters*SF_p_*and normalized*AUC*as they are (1) they are the most robust to noise (seeSection 3.1), (2) can beused as serve as a useful readout of the whole CSF (e.g., (Applegate et al., 1997)), and(3) can be consistently derived across a range of CSF parameterizations (Jigo et al., 2023).

**Fig. 5.**
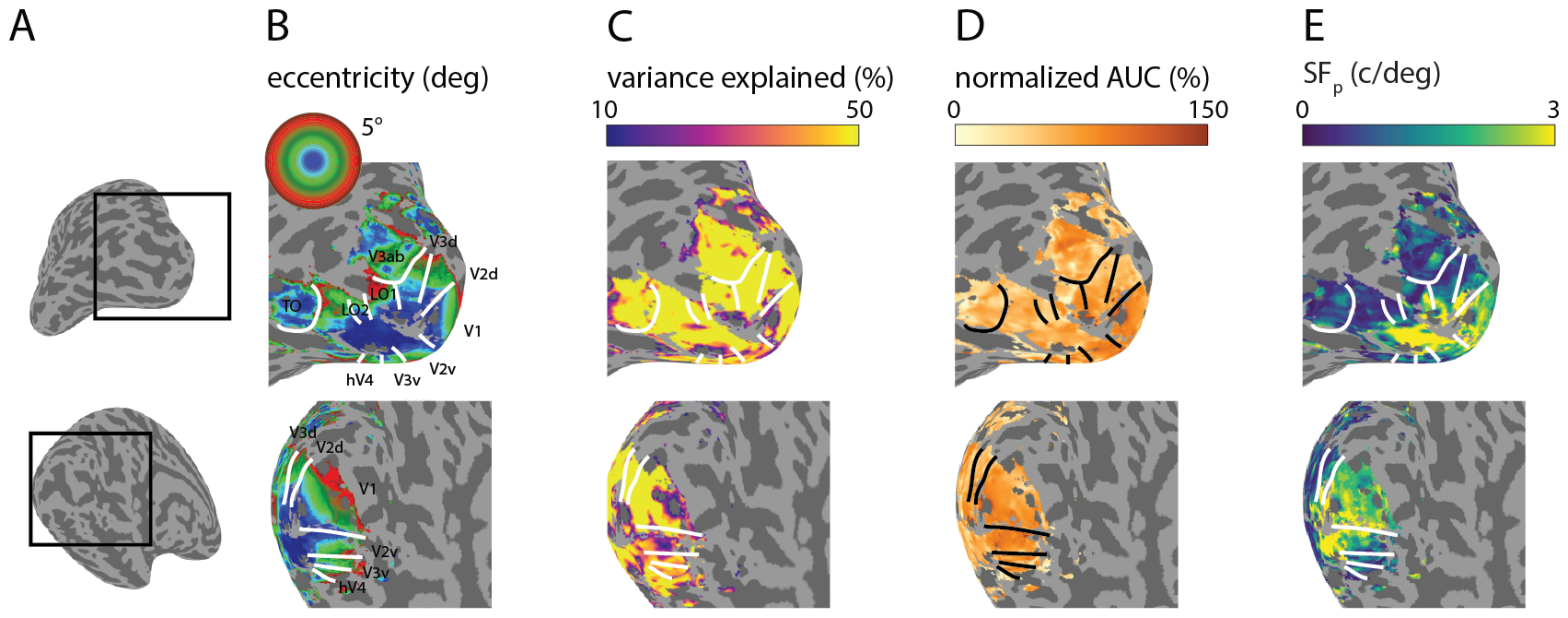
nCSF model parameters displayed on the cortical surface. Parameters areshown for participant 3 (seeFig. S8showing allparticipants’ surfaces). We included cortical locations withvariance explained >10% for visualization. (A) Inflated corticalsurface (left hemisphere) with the lateral (top row) and medial (bottomrow) views. Black boxes indicate the zoomed views for panels (B-E). (B)Eccentricity (deg, from pRF mapping data) for comparison with nCSFparameters. Borders of visual areas (white lines) are displayed (V1, V2,V3, V3ab, hV4, LO, TO). (C) Variance explained(*r^2^*%) of nCSF fits is high acrossvisual areas and eccentricities. (D) Normalized*AUC*nCSF model (%, output variable). (E) nCSF model parameter:*SF_p_*(c/deg).

The visual areas and eccentricity maps derived from pRF mapping for oneparticipant are shown on the inflated cortical surface in the region near theoccipital pole (Fig. 5, seeFig.S8for the other participants). The eccentricity maps range from0–5 degrees of visual angle, indicating the visual field representation.nCSF parameter and outcome estimates are projected on the inflated corticalsurface; seeFigure 5C-E.

The variance explained of the nCSF model is high across eccentricities and visualareas; seeFigure 5C. Thenormalized*AUC*is high around the foveal region and generallydecreases with eccentricity (Fig.5D).*SF_p_*is also higher near the fovealregion compared to the parafoveal region and decreases as a function ofeccentricity (seeFigs. 5Eand6B), consistent withprevious studies (Aghajari et al.,2020;Broderick et al.,2022;Henriksson et al.,2008). We observe that these systematic changes are similar betweenvisual areas and participants (seeFig. S6).

**Fig. 6.**
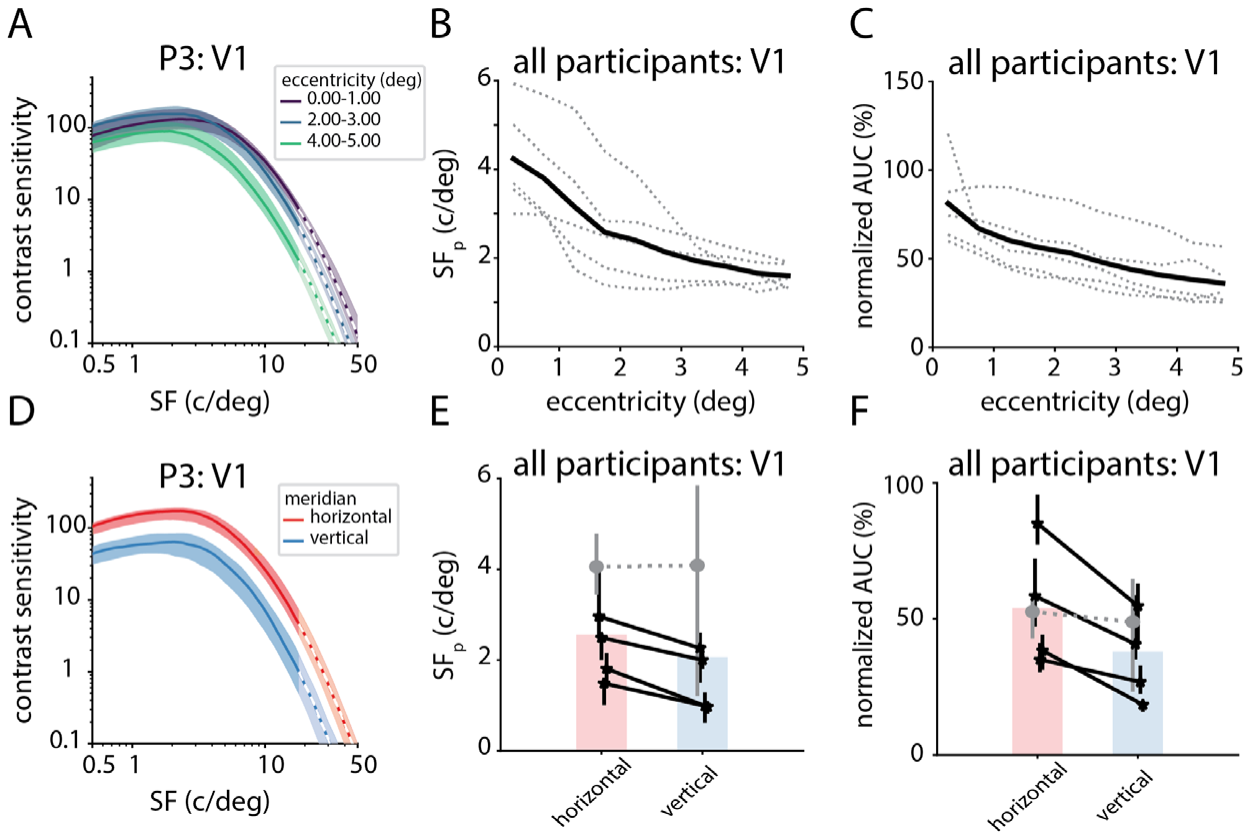
Variation in nCSF properties across eccentricity and polar angle withinV1. (A) nCSF curves in V1 for one participant, split by eccentricitybands (from pRF mapping: 0–1 degrees, 2–3 degrees,4–5 degrees eccentricity; represented by purple, blue and greenlines, respectively). Solid lines represent the median nCSF for allcortical locations within V1 and eccentricity band (where*r^2^*> 30% in both pRF and nCSFmodel fits). Shaded regions represent the 25th and 75th percentile ofthe nCSF curves. We extrapolated the nCSF beyond the range of spatialfrequencies present in the stimuli (>18 c/deg); where this hasbeen done, the lines become dotted and the shading becomes lighter. (B)and (C)*SF_p_*and normalized*AUC*decrease with eccentricity respectively acrossall participants in V1 (mean = thick black line, individuals= dotted gray lines); lines are the mean value binned byeccentricity (bin width = 0.5 deg). (D) nCSF curves for thehorizontal and the vertical meridian. Solid lines represent the mediannCSF for all cortical locations within the horizontal or verticalmeridian; shaded regions represent the 25th and 75th percentile of thenCSF curves. We extrapolated the nCSF beyond the range of spatialfrequencies present in the stimuli (>18 c/deg); where this hasbeen done, the lines become dotted and the shading becomes lighter. (E)and (F)*SF_p_*and normalized*AUC*for the horizontal and the vertical meridian.The solid lines indicate a significant difference within a participantbetween the horizontal and vertical meridian. The dotted lines indicatethat there is no significant difference between the horizontal andvertical meridian within a participant.

### 3.4 nCSF properties vary around the visual field in V1

To determine how nCSF parameters vary with eccentricity in V1, we used a linearregression analysis to fit the slope of eccentricity versus nCSF parameters.This analysis was performed separately per participant, only including verticeswhere both the nCSF and pRF model fits had a variance explained >30%. Tocorrect for volume-to-surface upsampling, we determined the degrees of freedomused in calculating the t-statistics by taking the number of cortical locations(vertices) divided by the upsampling factor.

Overall, a decrease in*SF_p_*and normalized*AUC*can be observed in the nCSF curves for differenteccentricity bands (Fig. 6Afor an example participant;Fig. S6for all participants), displaying ashift leftward (toward lower spatial frequencies) as well as a general decreasein the area under the curve.*SF_p_*decreases as afunction of eccentricity in V1 for all participants,*p*<0.001 (seeFig. 6BandTable S2).The normalized*AUC*also decreases with eccentricity in V1 forall participants,*p*< 0.001 (seeFig. 6CandTableS2).

We also compared how*SF_P_*and normalized*AUC*differ from the horizontal and vertical meridian byperforming an independent-sample t-test, separately per participant (again onlyincluding vertices where both the nCSF and pRF model fits had a varianceexplained >30% and correcting for volume-to-surface upsampling). Verticeswere assigned to the horizontal or vertical meridian if they were within 15(radial) degrees of the meridian, and had eccentricity values between 1 and 5degrees (to avoid ambiguous polar angles around the fovea, followingHimmelberg et al., 2022).Additionally, we found different nCSF curves for the horizontal and the verticalmeridian (seeFig. 6D, exampleparticipant), and lower values for*SF_P_*andnormalized*AUC*in the vertical meridian compared to thehorizontal meridian (*p*< 0.001 for all participantsexcept P1, seeFig. 6E,F).

### 3.5 nCSF properties vary across the cortical hierarchy

First, we compared nCSF curves across the cortical hierarchy. The nCSF curves aredifferent across the cortical hierarchy, for example between V1 and TO. This isexemplified inFigure 7A,which shows the nCSF curves for one participant (for a comprehensive view of allparticipants and visual areas, seeFig. S6). Notably, the center of the nCSFcurve for TO is further left (lower*SF_p_*) than V1.Next, we compared*SF_p_*and normalized*AUC*across eccentricity and the cortical hierarchy.Extrastriate regions tend to show a decrease in*SF_p_*and normalized*AUC*with eccentricity, similar to V1 (seeFig. 7B,7Cfor an example participant, andFig. S7for all other participants). However, this effect is less pronounced inextrastriate regions compared to V1. Additionally, there is an overall decreaseacross participants in*SF_p_*and*AUC*moving up the cortical hierarchy (seeFig. 7E,7F).Lastly, we investigated the*goodness-of-fit*of the nCSF model.Across all visual areas and participants, the nCSF model effectively capturesthe variance in the fMRI data (variance explained > 30%); seeFigure 7D.

**Fig. 7.**
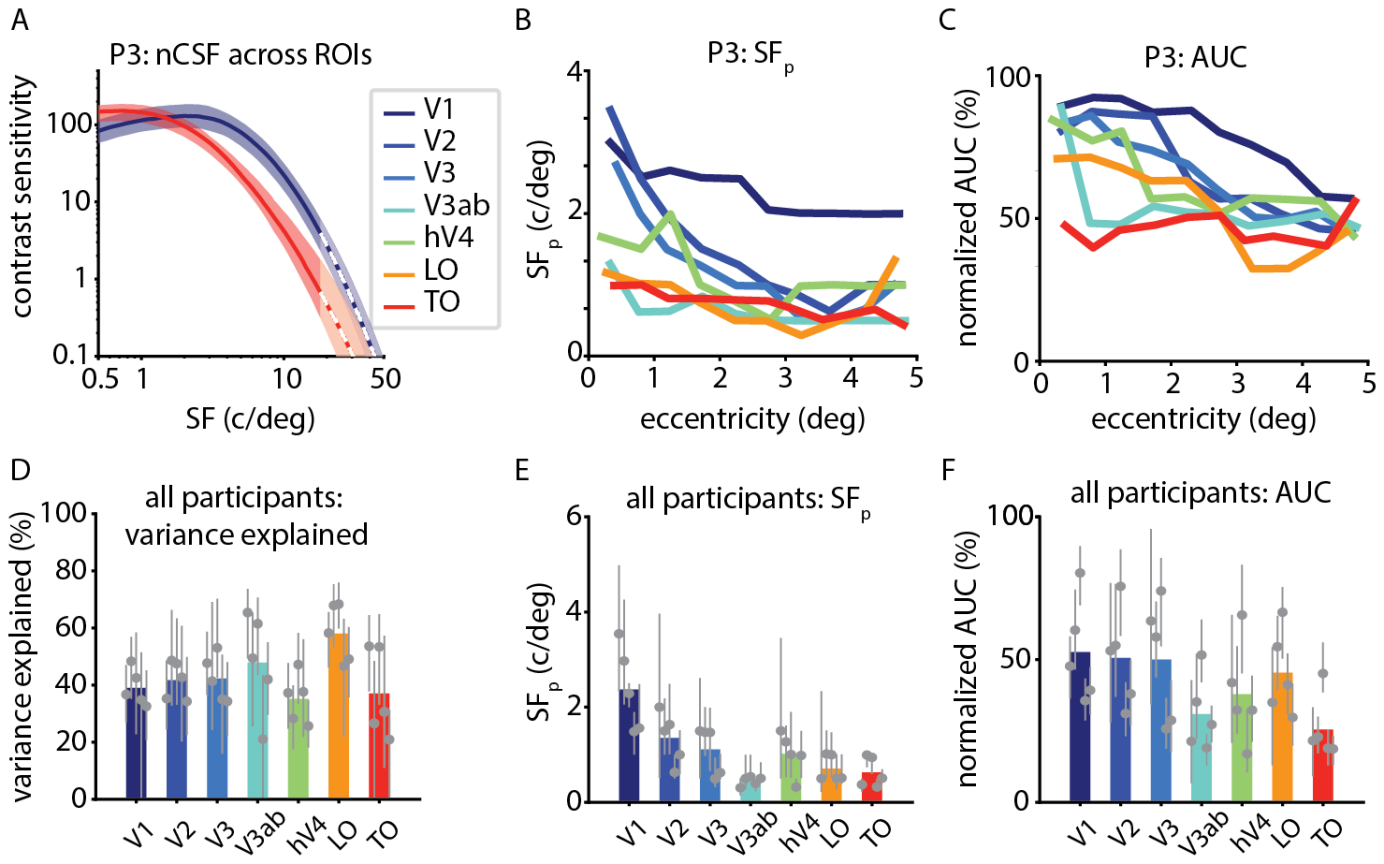
Variation in nCSF properties across the cortical hierarchy. (A) nCSFcurves in V1 (blue) and TO (red) for one participant. Solid linesrepresent the median nCSF for all cortical locations within a givenvisual area and eccentricity band (where*r^2^*> 30% in both pRF and nCSF model fits). Shaded regions representthe 25th and 75th percentile of the nCSFs. We extrapolated the nCSFbeyond the range of spatial frequencies present in the stimuli(>18 c/deg); where this has been done, the lines become dottedand the shading becomes lighter. (B) and (C)*SF_p_*and normalized*AUC*vary with eccentricity for one participant, across visual areas (linecolors). (D, E, and F) mean values for variance explained,*SF_p_*and normalized*AUC*across visual areas. Gray dots and barsindicate the median and interquartile range for individualparticipants.

## 4 Discussion

We introduce the neural contrast sensitivity function (nCSF) which is both a conceptand a method. As a concept, the nCSF describes the sensitivity of neural populationsas a function of spatial frequency and contrast. As a method, we model the nCSF inthe human visual cortex using fMRI and using an approach similar to pRF modeling(Dumoulin & Wandell,2008). The nCSF parameters were estimated from the fMRI data using abiologically-inspired, model-based approach similar to the pRF method. We model nCSFproperties using a combination of the CSF (Chung & Legge, 2016) and CRF (Boynton et al., 1999).

We validated the nCSF method using simulations, showing that the key parameters ofinterest—spatial frequency peak and area under the curve—can beestimated accurately and are robust to noise. Importantly, the nCSF modeleffectively captured the variance in the fMRI data across the visual cortex.Consistent with prior fMRI studies (Aghajari et al., 2020;Goulet & Farivar, 2024;Henriksson et al., 2008), we found that the peak spatialfrequency decreases as a function of eccentricity. In addition, total sensitivity(i.e., area under the curve) decreases with eccentricity. This effect was similaracross the visual cortex, but more pronounced in the early visual cortex (V1, V2,V3) versus the late visual cortex (e.g. LO and TO). We also find that peak spatialfrequency and area under the curve are lower in the vertical than horizontalmeridian which is in line with findings in psychophysics (Himmelberg et al., 2020,^2022^).

The nCSF concept combines notions of ophthalmology (Campbell & Gubisch, 1966;Le Grand, 1935;Michael et al., 2011), singleneuron recordings (Albrecht &Hamilton, 1982;Levitt etal., 1994;Sclar et al.,1990), and neuroimaging (Aghajari et al., 2020;Boynton et al., 1999;Henriksson et al., 2008). In ophthalmology, the nCSF refers toprocessing in the retina and visual hierarchy but independent of opticalaberrations. Though we measure brain responses directly, optical aberrations maystill contribute. However, different nCSF properties in the same visual fieldlocation across the different visual field maps cannot be attributed to opticalaberrations. Similarly, variations across eccentricity and polar angle are notlikely due to optical aberrations, but due to known neural processing differences.Our nCSF concept also differs from single neuron recordings. For example, apopulation of neurons and the hemodynamic response properties contribute to thesignal. These considerations are similar to the considerations underlying theconcept of the pRF (Dumoulin &Knapen, 2018;Dumoulin& Wandell, 2008). We measure the nCSF using fMRI, but, inprinciple, the nCSF is independent of the measurement modality and can also bemeasured with other imaging techniques, such as invasive human electrodes (Harvey et al., 2013;Yoshor et al., 2007) or MEG (Eickhoff et al., 2024;Kupers et al., 2021).

Many factors may lead to changes in nCSF properties, for example using differentstimuli or tasks. Different stimuli, for example different temporal characteristics,may elicit responses from different neural populations and therefore result indifferent nCSF. Here, we used gratings, but the same nCSF model could be fit andapplied with any stimuli which covers the range of spatial frequencies and contrastsof interest; allowing comparisons across a large range of potential experiments.Likewise, attention influences the behavioral CSF, and therefore different tasks mayinfluence the nCSF (Cameron et al.,2002;Jigo &Carrasco, 2020). Thus, the stimuli and tasks are likely to influence theneural population driving the responses underlying the nCSF model.

Furthermore, the nCSF model is based on a psychophysical model of the CSF, which canbe parameterized using many different functions. We selected the asymmetriclogarithmic parabolic function (aLP,Chung & Legge, 2016); fixing the width of the left side of thefunction, giving a 3 parameter model (*CS_p_, SF_p_,width_r_*). We selected this model as a balance betweenflexibility and simplicity. The advantage of the aLP over symmetrical models is thatit can capture the slower decrease in sensitivity to lower spatial frequenciescompared with the more rapid decrease toward higher spatial frequencies, with thesame number of parameters. Furthermore, disorders affecting the CSF typically (butnot always, see below) affect it in characteristic ways, with either (1) a uniformdrop in contrast sensitivity (translation down the y axis), (2) uniform drop inspatial frequency (translation leftward), or (3) selective sensitivity loss at highspatial frequencies (Chung &Legge, 2016). The aLP can appropriately capture all these changes, with asmall number of parameters.

However, alternative CSF equations would likely produce similar results (Watson & Ahumada, 2005).Jigo et al. (2023)foundthat of the nine different functions, the “YQM” model (fromYang et al., 1995) provided thebest fit to the data, but the aLP and several other models performed similarly.Previous fMRI studies of spatial frequency sensitivity have included a log-gaussian(Aghajari et al., 2020) and asymmetrical log parabolic function (Goulet & Farivar, 2024). Given the similarities between thefunctions (Watson & Ahumada,2005), it is likely that these different models will perform similarly,especially on core parameters such as peak spatial frequency and total sensitivity(area under the curve).

It is likely the optimal form of the model will vary depending on use case. For someconditions, a two-parameter model may be appropriate (Chung & Legge, 2016). In some specific patientgroups, a more sophisticated model will be necessary, for example selective loss ofsensitivity at low spatial frequencies (cataracts) or selective loss at middlespatial frequencies (multiple sclerosis) would not be captured well by thethree-parameter form of the aLP.

Just as there are alternative functions to describe the CSF component, there arealternative functions to describe the CRF. Here, we used a modified form of theNaka-Rushton function, with a single slope parameter allowing us to capture thenonlinear responses to increases in contrast. Many alternative functions have beenused to fit the CRF, including the two-parameter form the Naka-Rushton function(Boynton et al., 1999) aswell as linear, logarithmic, and exponential functions (e.g.,Albrecht & Hamilton, 1982)and a binary step function (Goulet& Farivar, 2024). We selected the Naka-Rushton function as it isoften used to model the CRF and is biologically plausible, approximating the gaincontrol known to occur in cortical neurons (Heeger, 1992). For fMRI data, there is evidence thatsustained adaptation promotes nonlinearities in the CRF, while unadapted responseswill be more linear (Vinke et al.,2022). Hence, in some conditions, a linear model might be moreappropriate, depending on the duration of the stimulus. Similarly, using stimuliwhich move more slowly through contrast space will likely result in more accurateestimates for the CRF (see CRF Simulation and Estimating the*slope_CRF_*sections inSupplementaryMaterials).

In summary, our implementation of the nCSF offers a robust and accurate fit to thedata while maintaining simplicity and interpretability. However, it is not the onlypossible implementation. In the same way that the pRF model (Dumoulin & Wandell, 2008)has been extended, we expect that the nCSF model will also be adapted and refinedfor different applications. Future models could benefit from integrating establishedcomputational motifs such as suppression (Zuiderbaan et al., 2012), response compression (Kay et al., 2013), and divisivenormalization (Aqil et al., 2021)to enhance their predictive power. In studies involving patient populations wherethe shape of the CSF may vary or be less predictable, model-free approaches could beespecially valuable for capturing unique characteristics and providing a moreflexible framework (Carvalho et al.,2020;Greene et al.,2014;Lee et al.,2013).

The psychophysical CSF is widely used to assess perception in health and disease(Chung & Legge, 2016;Hou et al., 2010;Pelli & Bex, 2013).However, neural CSFs can vary across different cortical regions and levels of theprocessing hierarchy, and these variations may not always align with perceptualCSFs. This discrepancy highlights the need to investigate both perceptual and neuralmeasures, as deficits might be present in one domain but not the other. The nCSFmodel provides a useful starting point for future research in this area, helping tobetter understand the diverse characteristics of neural computations acrossdifferent contexts, populations, and conditions.

## 5 Conclusion

We introduce a novel method to measure the nCSF in the human visual cortex. Weprovide a quantitative, validated framework and show how nCSF properties vary acrossvisual areas and with eccentricity. This method can be applied to both healthy andclinical conditions, and provide novel insights into the cortical organizationunderlying perception.

## Supplementary Material

Supplementary Material

## Data Availability

The code generated for this paper is available here:https://github.com/spinoza-centre/prfpy_csenf The data are considered personal data pursuant to the General Data ProtectionRegulation (GDPR) and can only be shared based on and subject to the policies of theRoyal Netherlands Academy of Arts and Sciences (KNAW). The datasets are availablefrom the corresponding author on request. Considering the requirements imposed bylaw and the sensitive nature of personal data, any requests will be addressed on acase-by-case basis, subject to a data usage agreement.
