# Supplementary material for: Modeling neural contrast sensitivity functions in human visual cortex
Source: Imaging Neurosci (Camb). 2025 Feb 18;3:imag_a_00469. doi: 10.1162/imag_a_00469 (PMC12319964; doi:10.1162/imag_a_00469)
Supplement: Supplementary Material [file imag_a_00469-supp.docx]

Supplementary Materials

| SF (c/deg) | Michelson contrast (%) | | | | | | | | | | | |
| --- | --- | --- | --- | --- | --- | --- | --- | --- | --- | --- | --- | --- |
| 0.5 | 0.25 | 0.39 | 0.61 | 0.95 | 1.48 | 2.30 | 3.58 | 5.58 | 8.70 | 13.56 | 32.94 | 80.00 |
| 1 | 0.25 | 0.39 | 0.61 | 0.95 | 1.48 | 2.30 | 3.58 | 5.58 | 8.70 | 13.56 | 32.94 | 80.00 |
| 3 | 0.25 | 0.39 | 0.61 | 0.95 | 1.48 | 2.30 | 3.58 | 5.58 | 8.70 | 13.56 | 32.94 | 80.00 |
| 6 | 0.25 | 0.61 | 0.95 | 1.48 | 2.30 | 3.58 | 5.58 | 8.70 | 13.56 | 21.13 | 32.94 | 80.00 |
| 12 | 0.25 | 0.61 | 1.48 | 2.30 | 3.58 | 5.58 | 8.70 | 13.56 | 21.13 | 32.94 | 51.33 | 80.00 |
| 18 | 0.25 | 0.61 | 1.48 | 2.30 | 3.58 | 5.58 | 8.70 | 13.56 | 21.13 | 32.94 | 51.33 | 80.00 |

###

### **Table S1. Stimulus contrast conditions.** In each stimulus block gratings with the same spatial frequency (0.5, 1, 3, ,6, 12, or 18 c/deg) are shown but different Michelson contrast values. The contrast range presented was dependent on the spatial frequency shown in the stimulus block, ensuring an optimal sampling of the CSF (see Figure 1C).

*HRF simulation*

We used a default two-gamma HRF with five parameters: *time to peak* gamma 1, *full width at half maximum* (*FWHM*) gamma 1, *time to peak* gamma 2, *FWHM* gamma 2, *dip* (Friston et al., 1998; Glover, 1999). The standard values are set at *FWHM* gamma 1 = 5.4 s, *time to peak* gamma 1 = 5.2 s, *FWHM* gamma 2 = 10.8 s, *time to peak* gamma 2 = 7.35 s, *dip* = 0.35 s. We generated 50 HRFs by randomly selecting values for each of the five parameters. Each parameter was allowed to deviate from their standard value by a maximum of 30%, see Figure S1.

A combination of nCSF model parameters was chosen (*SF_p_* = 1 c/deg, *CS_p_* = 150 (a.u.), *width_R_* = 1.3 (a.u.), *slope_CRF_* = 1.5 (a.u.), see green curve in Figure 3) and a synthetic dataset was generated containing 100 voxel time series. We fitted the nCSF model to the synthetic dataset with each time a different HRF. The resulting nCSF estimates (normalized *AUC* , *SF_p_*, *CS_p_*, *slope_CRF_*) as a function of *FWHM* and *time to peak* are shown in Figure S2. Normalized *AUC*, *SF_p_* and *slope_CRF_* remain relatively constant when changing the *FWHM* and *time to peak* (see Figure S2 panels A, B, D, E, F, H). However, *slope_CRF_* is systematically overestimated for both HRF parameters, indicating a more binary response. *CS_p_* is decreasing with an increase in *FWHM* (see Figure S2C), but is relatively constant when changing time to peak (see Figure S2G). Overall, some parameters, in particular normalized *AUC* and *SF_p_*, are more stable than others when changing *FWHM* and *time to peak* (in particular *CS_p_*).


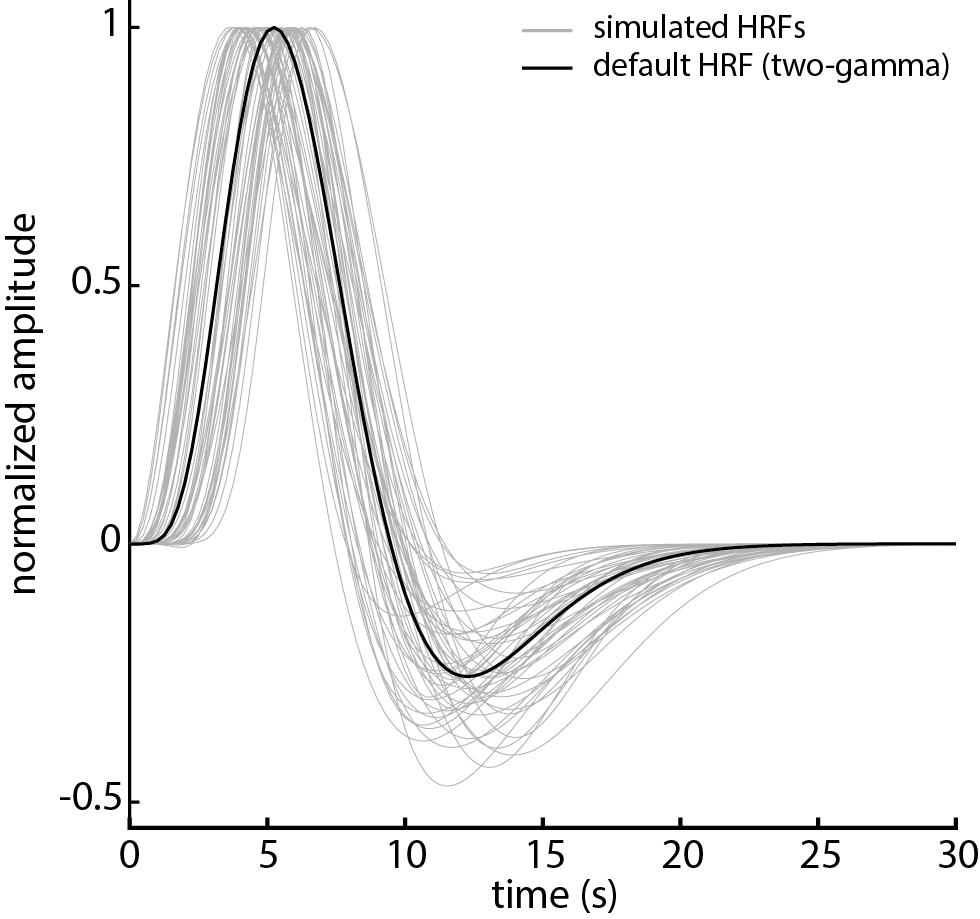


**Figure S1. Simulated HRFs.** The different HRFs used to estimate the nCSF model parameters. We generated 50 two-gamma HRFs by randomly selecting values for each of the five parameters, each parameter was allowed to deviate from their standard value by a maximum of 30%. The black line indicates the default two-gamma HRF [(Glover, 1999)](https://www.zotero.org/google-docs/?tJKNHL), the gray lines represent the generated HRFs.

### **
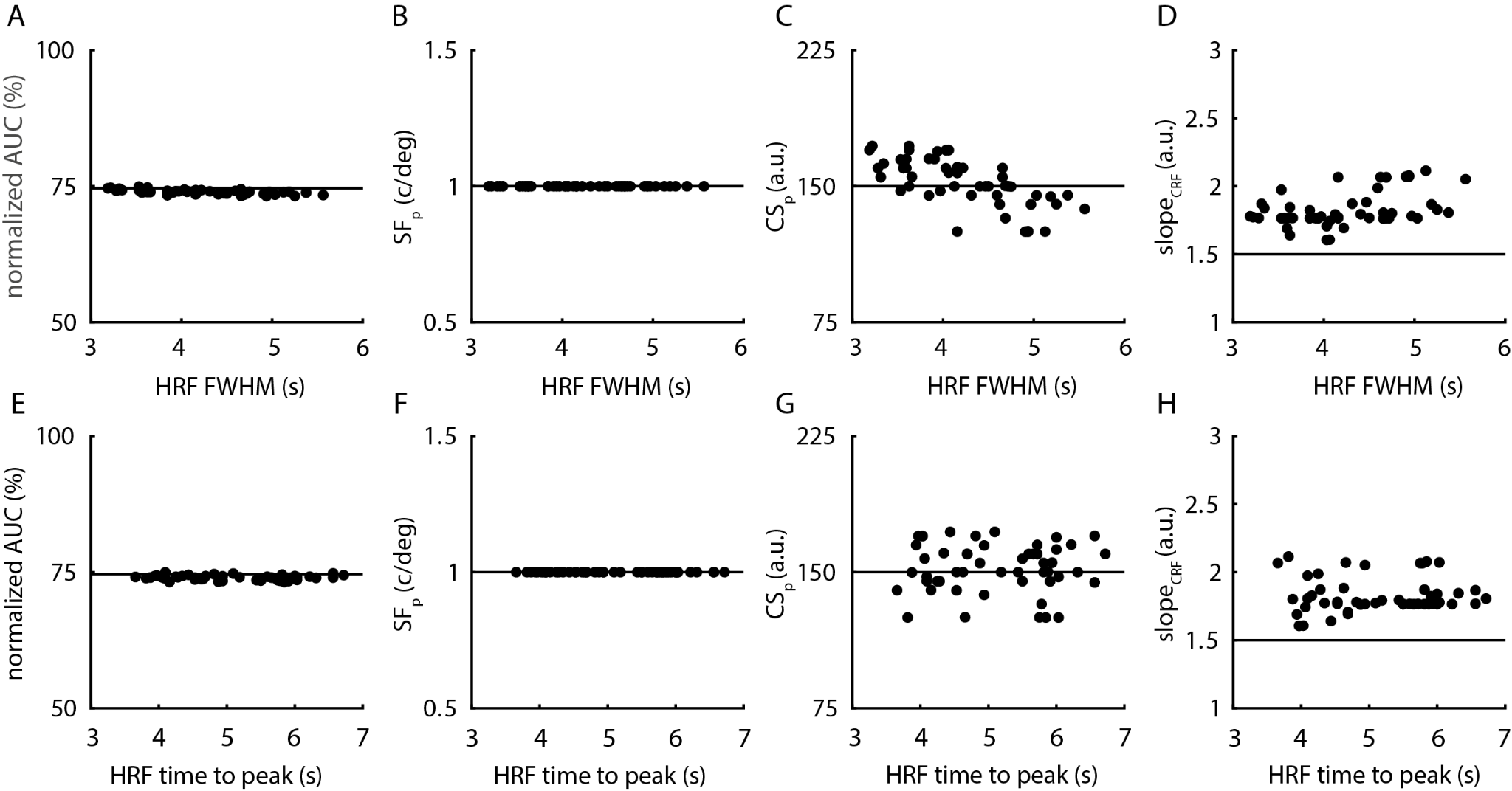
**

### **Figure S2. The relationship between the HRF model parameters and the nCSF model parameters.** The effect of the HRF parameters (*FWHM* gamma 1 and *time to peak* gamma 1) on the nCSF model estimates: normalized *AUC* , *SF_p_* , *CS_p_* , *slope_CRF_*). The line indicates the chosen value of the synthetic dataset. The dots represent the median values of 100 voxel time series. A) The effect of normalized *AUC* (c/deg) on *HRF FWHM* (s). B) The effect of *SF_p_* (c/deg) on *HRF FWHM* (s). C) The effect of *CS_p_* on *FWHM* (s). D) The effect of *slope_CRF_* on *HRF FWHM* (s). E) The effect of normalized *AUC* (c/deg) on *HRF time to peak* (s). F) The effect of *SF_p_* (c/deg) on *HRF time to peak* (s). G) The effect of *CS_p_* on *HRF time to peak* (s). H) The effect of *slope_CRF_* on *HRF time to peak* (s).

*CRF simulation*

fMRI data were collected using stimuli varying in spatial frequency (6 levels) and contrast (14 levels), stepping in contrast with each TR (1.5 s), see *Methods, 2.2 Stimulus presentation*. Simulations show that these stimulus conditions are adequate for recovering the nCSF parameters of interest (*SF_p_*, normalized *AUC*). However, these stimulus conditions are not optimal for recovering the *slope_CRF_*, since we are only able to accurately capture the CRF (and associated parameter *slope_CRF_*) when noise is very low (see Figure S3A-C).

*
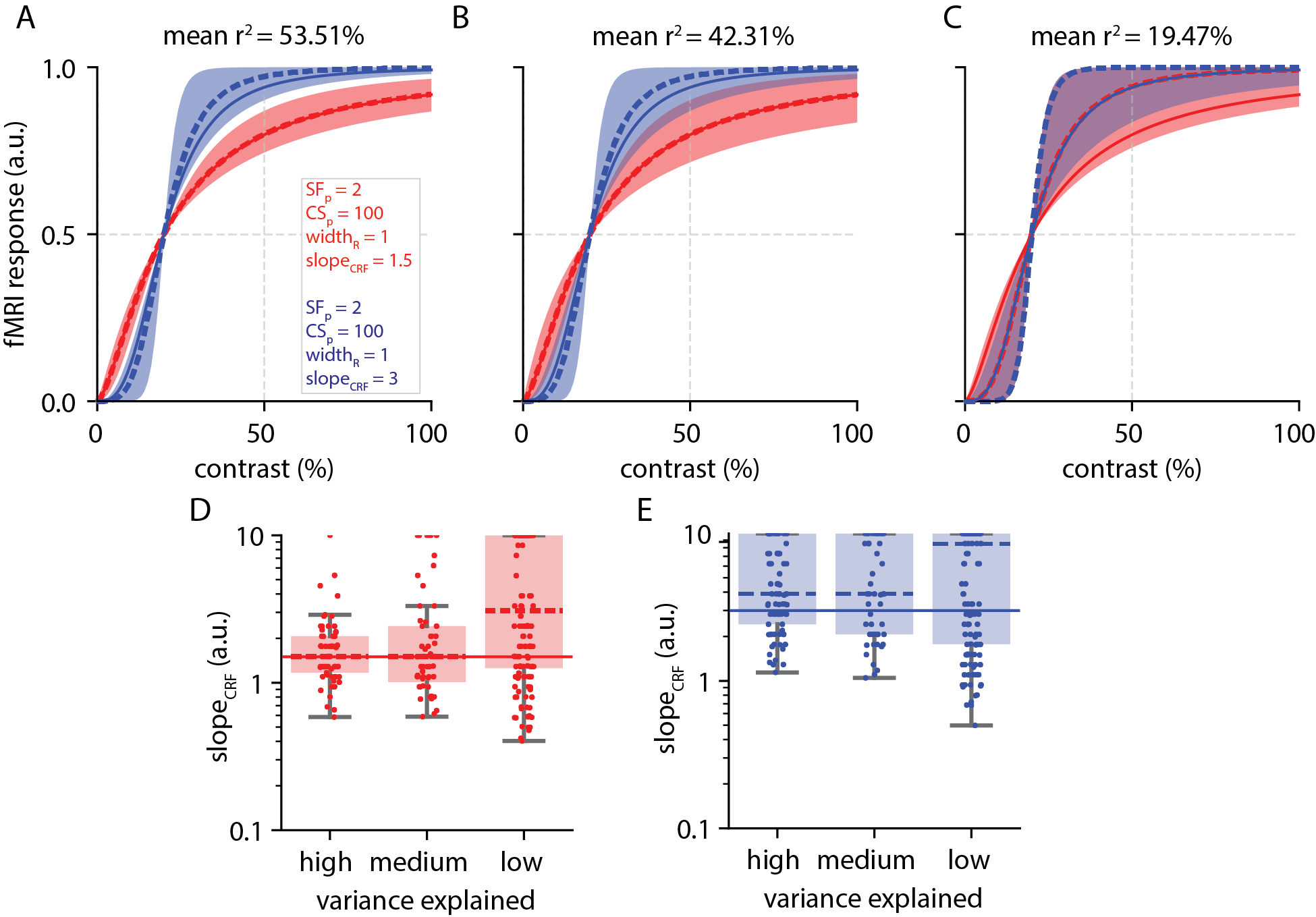
*

### **Figure S3**. **CRF simulations across different noise levels.** Simulations, as in the main text, but here varying only the *slope_CRF_* parameter (purple = 1.5, light blue = 3). For each combination of parameters, the results are shown for synthetic data (100 permutations) and three variance explained categories (high: *r^2^* > 50%; medium: 30% < *r^2^* < 50%; and low: 10% < *r^2^* < 30%, left to right, respectively). The solid lines represent the CRF based on the chosen parameters, the dashed lines represent the median CRF, the shaded areas represent the 25th and 75th percentile. Panels D-E show how well the parameters are recaptured: solid lines indicate the true nCSF model parameters, whereas the dashed lines and distributions represent the median values of 100 permutations.

We then re-ran the simulations varying the speed at which the stimulus changed contrast (i.e., the number of contrast levels in the stimulus). It is possible to accurately capture the CSF parameters, with even fewer contrast levels than was used in the experiment (see Figure S4A versus Figure S4B-C). However, with fewer contrast levels, it becomes impossible to capture the CRF even if there is very little noise (S4D-F); and the corresponding *slope_CRF_* parameter becomes unstable (S4G-H). If the *slope_CRF_* is of interest, we recommend increasing the stimulus time, by adding more levels in the contrast dimension (or alternatively spending longer at each level). However, if the primary interest is in the CSF parameters (*SF_p_* etc), then the stimuli can be sped up by sampling the contrast dimension more sparsely. We anticipate that this will be particularly useful in patient populations where the duration of the scan should be as short as possible.


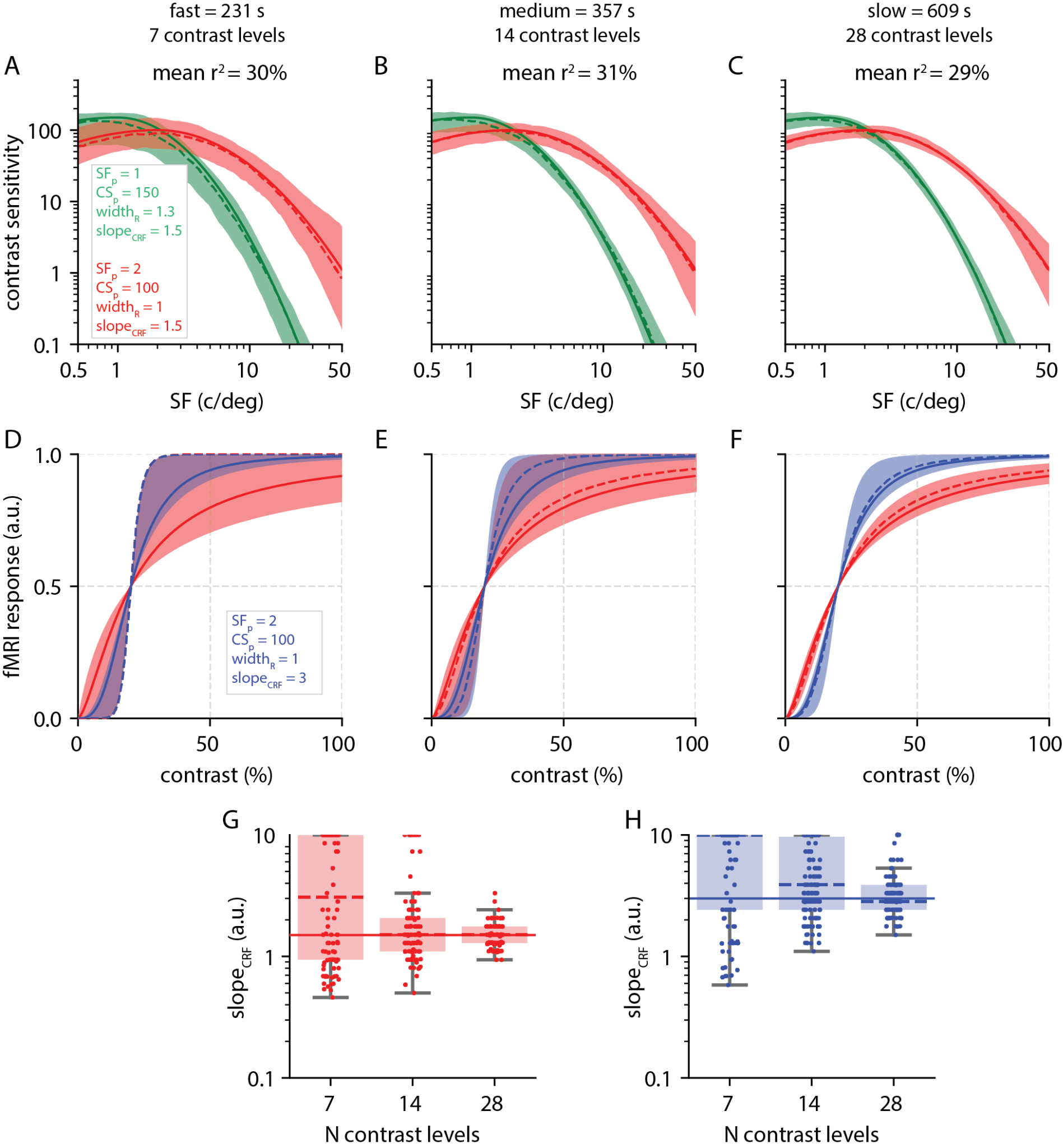


### **Figure S4**. **Simulations with moving at different speeds through contrast space.** As above, but here the speed at which the stimulus changes contrast (fast: 7 contrast levels = 231 s/run; medium: 14 contrast levels = 357 s/run; slow: 28 contrast levels = 609 s/run) varies (noise levels are held constant). A-C) nCSF recovered for 7, 14 and 28 contrast levels, respectively. D-F) CRF recovered with a different number of steps in the contrast dimension. G-H) stability of the *slope_CRF_* parameter with different numbers of contrast levels.

*Estimating the slope_CRF_*

The CRF allows for a smooth (non-binary) transition in sensitivity, determined by the parameter *slope_CRF._*. Estimates of *slope_CRF_* did not follow predictions from previous literature, i.e., lower in V1, and higher in later cortical regions (TO). We expect that this is a limitation of the stimulus design (see *CRF simulation*). In future studies, if the stimulus moves through contrast space at a fast speed, we suggest fixing the *slope_CRF_* parameter to 1.70 (mean *slope_CRF_* value, across participants and ROIs, weighted by variance explained).

### **
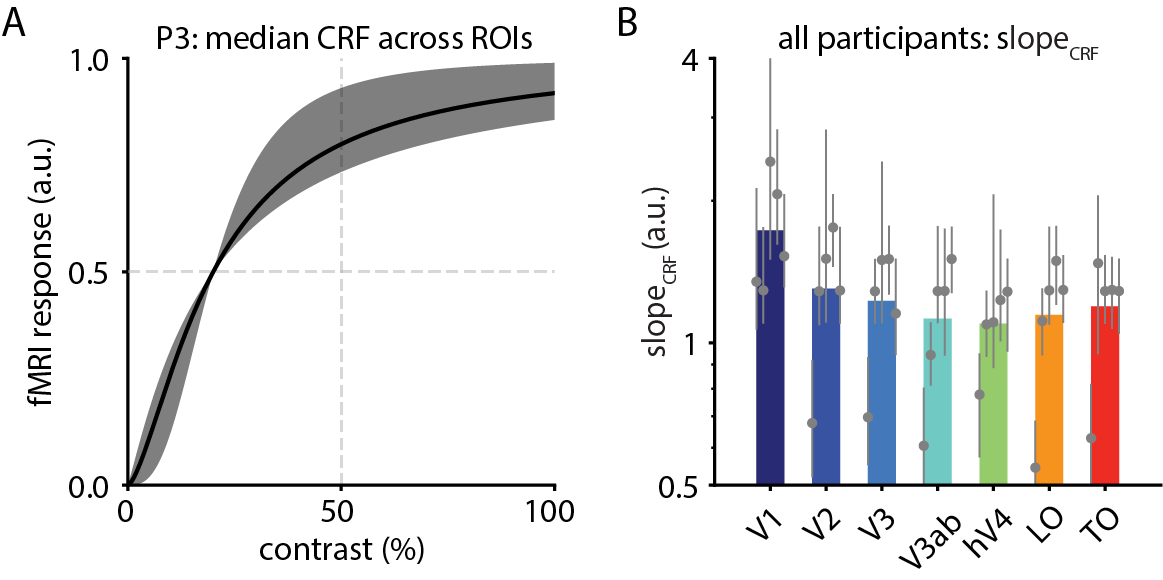
**

### **Figure S5**. **CRF estimated across participants and ROIs.** A) Displays an example CRF curve for participant 3, taken across the whole cortex Solid line indicates the median, shaded region indicates the 25th and 75th percentiles. B) Shows how *slope_CRF_* varies across the visual hierarchy. Across all participants and ROIs the mean *slope_CRF_* value, weighted by variance explained was 1.70.

### *
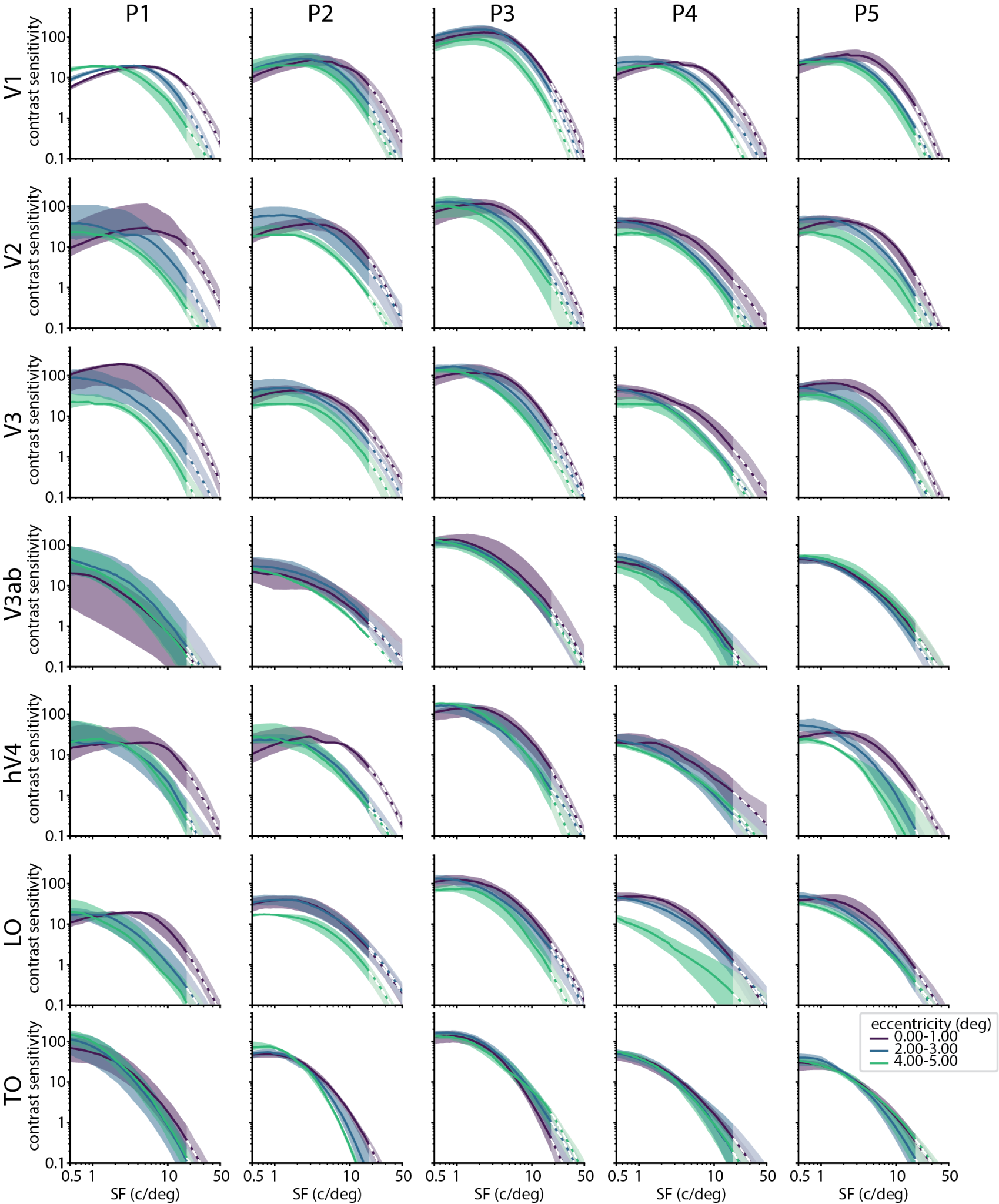
*

### **Figure S6**. **nCSF fits across eccentricities for all participants and ROIs.** nCSF fits are split by eccentricity band (from pRF mapping: 0-1 degrees, 2-3 degrees, 4-5 degrees eccentricity; represented by purple, blue and green lines, respectively). Solid lines represent the median nCSF for all cortical locations within a given ROI and eccentricity band (where *r^2^* > 30% in both pRF and nCSF model fits). Shaded regions represent the 25th and 75th percentile of the nCSFs. We extrapolated the nCSF beyond the range of SFs present in the stimuli (>18 c/deg), where this has been done the lines become dotted and the shading becomes lighter.


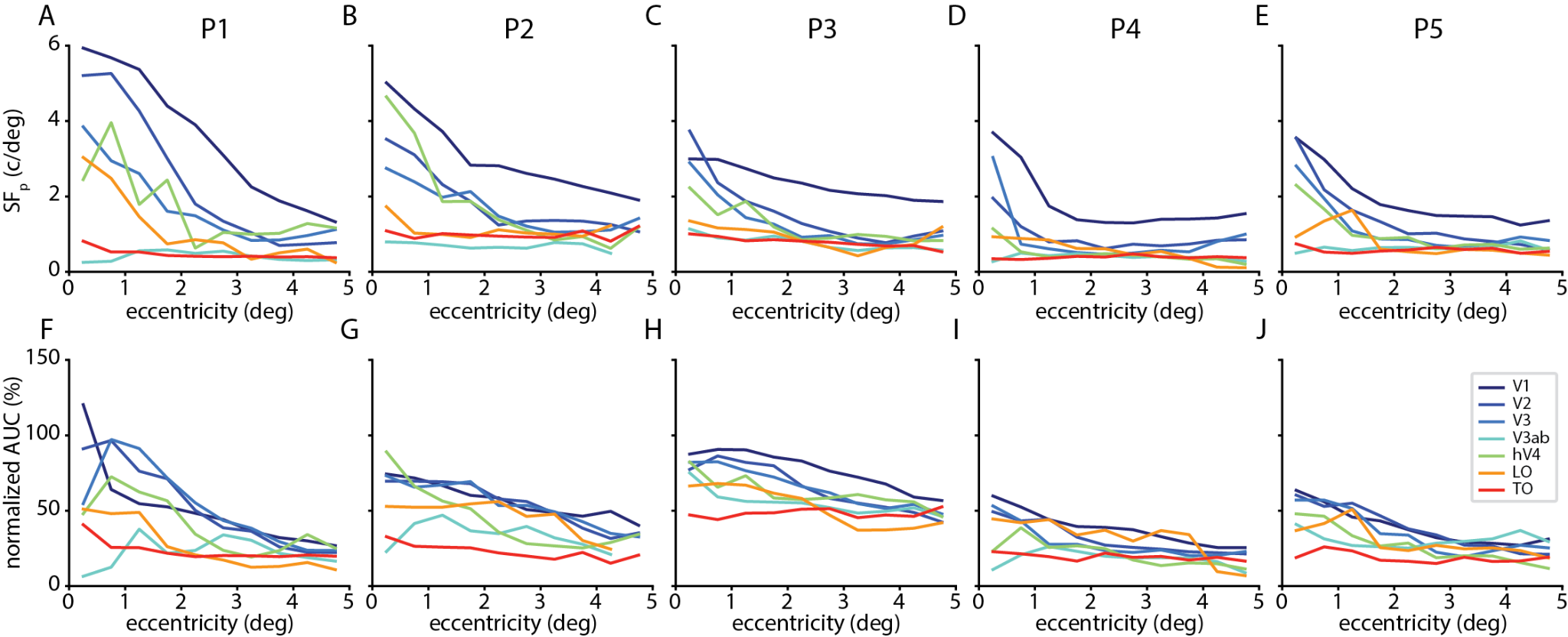


### **Figure S7. Variation in nCSF properties across eccentricity and the cortical hierarchy.** A-E) *SF_p_* (c/deg) across eccentricity (deg) and ROIs for all participants (P1-P5). F-J) Normalized *AUC* (%) across eccentricity (deg) and ROIs for all participants (P1-P5).

| slope of nCSF parameters with eccentricity in V1 | | | | | |
| --- | --- | --- | --- | --- | --- |
| parameter | participant | eccentricity slope | t-statistic | p-value | degrees of freedom (adjusted) |
| *AUC* (%) | P1 | -1.26 | -30.02 | <0.01 | 298.24 |
| *AUC* (%) | P2 | -11.86 | -8.43 | <0.01 | 298.24 |
| *AUC* (%) | P3 | -0.61 | -6.85 | <0.01 | 298.24 |
| *AUC* (%) | P4 | -0.68 | -12.15 | <0.01 | 285.86 |
| *AUC* (%) | P5 | -7.87 | -11.7 | <0.01 | 285.86 |
| *SF_p_* (c/deg) | P1 | -0.16 | -2.51 | 0.01 | 285.86 |
| *SF_p_* (c/deg) | P2 | -0.28 | -12.96 | <0.01 | 380.98 |
| *SF_p_* (c/deg) | P3 | -8.98 | -17.54 | <0.01 | 380.98 |
| *SF_p_* (c/deg) | P4 | -1.12 | -7.63 | <0.01 | 380.98 |
| *SF_p_* (c/deg) | P5 | -0.27 | -8.52 | <0.01 | 502.89 |

### **Table S2. Results of linear regression of eccentricity on nCSF model parameters.** Parameters explored are *SF_p_* , and *AUC*. Models are fit separately per participant, only including V1 cortical locations with a high variance explained (>30% in both pRF and nCSF model fits). Degrees of freedom were obtained by taking the number of cortical locations in the ROI, and dividing by the volume-to-surface upsampling factor.

###
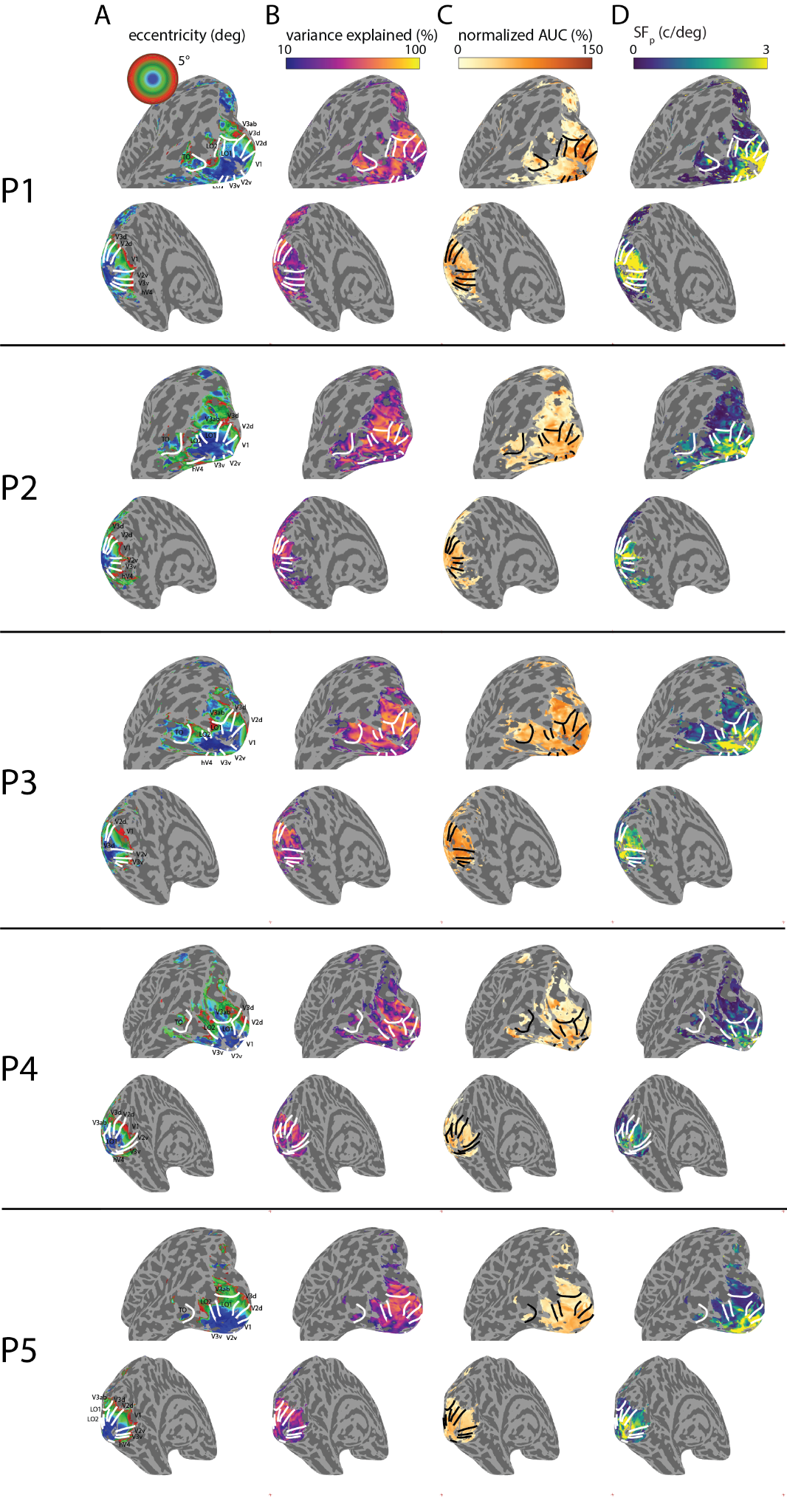


### **Figure S8. nCSF model parameters displayed on the cortical surface.** Parameters are shown for all participants. We included cortical locations with variance explained >10% for visualization. Inflated cortical surfaces (left hemisphere) and ROIs with the lateral (top row) and medial (bottom row) views. Eccentricity (deg, from pRF mapping data) for comparison with nCSF parameters. Borders of ROIs (white lines) are displayed (V1, V2, V3, V3ab, hV4, LO, TO). Variance explained (*r^2^* %) of nCSF fits is high across ROIs and eccentricities. Panels D-E display nCSF model parameters: D) Normalized *AUC* (%, output variable). E) *SF_p_* (c/deg).

References

Friston, K. J., Fletcher, P., Josephs, O., Holmes, A., Rugg, M. D., & Turner, R. (1998). Event-related fMRI: Characterizing differential responses. *Neuroimage*, *7*(1), 30–40.

Glover, G. H. (1999). Deconvolution of impulse response in event-related BOLD fMRI. *NeuroImage*, *9*(4), 416–429. https://doi.org/10.1006/nimg.1998.0419
